# 3D printed aluminum flat heat pipes with micro grooves for efficient thermal management of high power LEDs

**DOI:** 10.1038/s41598-021-87798-4

**Published:** 2021-04-15

**Authors:** Chao Chang, Zhaoyang Han, Xiaoyu He, Zongyu Wang, Yulong Ji

**Affiliations:** Institute of Marine Engineering and Thermal Science, Marine Engineering College, Dalian Maritime University, Dalian, 116026 People’s Republic of China

**Keywords:** Materials science, Engineering

## Abstract

As the electronic technology becomes increasingly integrated and miniaturized, thermal management has become a major challenge for electronic device applications. A heat pipe is a highly efficient two-phase heat transfer device. Due to its simple structure, high thermal conductivity and good temperature uniformity, it has been used in many different industrial fields. A novel aluminum flat heat pipe, with micro-grooves, has in the present work been designed and fabricated by using a 3D printing technology. Aluminum powder was used as a raw material, which was selectively melted and solidified to form the shape of the heat pipe. The sintered aluminum powder increased the roughness of the inner surface of the heat pipe, and the designed micro-grooves further enhanced the capillary forces induced by the wick structure. The wettability, for the working fluid (acetone), was excellent and the capillary forces were sufficient for the working fluid to flow back in the pipe. The effects of working fluid filling ratio, on the heat transfer performance of the heat pipe, was also investigated. It was shown that a filling ratio of 10% gave the best heat transfer performance with the lowest thermal resistance. The 3D-printed flat heat pipe was, therefore, also tested for the thermal management of a LED. The temperature of the LED could be kept within 40 °C and its service life became prolonged.

## Introduction

With the rapid development of the microelectronic technology, and the growing need by the information industries, the integration and miniaturization of electronic equipment have become a new development trend^1,2^. The electronic equipment has nowadays become more compact, and they are often designed for multiple functions. Many electronic components are then integrated into a single equipment, which most often results in serious heat dissipation problems^3,4^. An effective thermal management is, therefore, a key factor to ensure a stable operation of, e.g., high power electronic chips. To achieve an effective thermal control of various electronics, the heat that is generated by the electronic equipment is usually dissipated via heat pipe technology, phase change materials technology, spraying cooling technology or microchannel technology. Due to the simple structure, low cost and high thermal conductivity, the flat heat pipe has become one of the most common heat transfer devices for thermal management in various types of electronics^5,6–8^. A typical flat heat pipe is composed of a container, a working fluid and a wick structure. This wick structure is arranged on the inner surface of the pipe, and a small quantity of the working fluid is sealed into it (under partial vacuum). When heat is applied to the outer surface of the heat pipe, the working fluid inside will be heated and vaporized. The hot vapor will then quickly move to the condenser section of the pipe, where it is condensed to a liquid, thereby releasing the carried thermal energy. The condensed liquid will, thereafter, be pumped back to the evaporator section with the assistance of the capillary forces created by the wick structure. The whole thermodynamic cycle is, then, completed. Relying on the phase-change heat transfer and the two-phase flow, the flat heat pipe can transfer heat over long distances.

To date, heat pipes can be fabricated in many different types, including thermosyphons^9^, capillary heat pipes^10–12^, loop heat pipes^13,14^, miniature heat pipes^15^ and oscillating heat pipes^16–20^. Owing to their advantages of high thermal conductivity and low cost, capillary heat pipes have been widely applied within field likes electronics^21^, aerospace^22^, solar heating water systems^23–25^ and battery thermal management^26,27^. In the capillary heat pipe, the wick structure (as the most important component) not only provides a significantly efficient capillary force to pump condensed liquid back to the evaporator section, it also increases the heat transfer area of the evaporator and condenser sections, which will further affect the heat transfer performance of the heat pipe. Hence, the property of the wick structure plays a vital role for the performance of the heat pipe. Intensive efforts have earlier been made in the development of various types of wick structures, such as sintered powder^28,29^, micro-groove^30–33^, mesh^34,35^ and composite wick structures^36–39^. Sintered powder is a common wick structure for traditional heat pipes. It provides a large capillary force for the closed cycling of the working fluid, which is due to the small pore size and high liquid permeability. However, the sintered wick structure increases the thickness of the heat pipe, which leads to a high thermal resistance. On the contrary, a micro-grooved wick structure presents a low thermal resistance, but small capillary force, which limits its practical applications. Furthermore, the metallic mesh can be deformed and has been applied in many types of heat pipes (especially in flexible heat pipes). It has, however, problems with a low capillary force. Hence, a single type of wick structure cannot satisfy the requirement for both a low thermal resistance and a high capillary force. Composite wick structures have earlier been proposed to enhance the heat transfer performance of the heat pipes. For example, Tang et al.^40^ reported a novel sintered copper mesh wick structure for ultra-thick heat pipes, which was fabricated by using a combination of chemical deposition and a sintering process. This wick structure has a larger capillary force, as compared with a normal mesh wick structure. Xu et al.^41^ developed a flat heat pipe by combining a groove structure with a nanoflower structure. The CuO nanoflower was found to improve the wettability of the wick structure, and the thermal conductivity of the flat heat pipe became 17% larger than that for a flat heat pipe with only a groove structure.

In general, current flat heat pipes used for electronics are made of copper, which is a relatively heavy material^42–44^. To meet the requirements for a light weight material, in addition to miniaturization, most recent research interests have focused on aluminum heat pipes (with their operative temperature range and light weight)^45–48^. For example, Tang et al.^49^ designed a novel aluminum heat pipe with a micro V-grooved wick structure. It was fabricated by a ploughing-extrusion method and the inner surface was, thereafter, treated by chemical corrosion. The experimental results showed that a combination of the ploughing-extrusion process and the chemical corrosion method was a convenient and effective way to enhance the heat transfer performance of the heat pipes. Chen et al.^50^ fabricated a micro-grooved aluminum flat plate heat pipe by using a hot extrusion method, and the wick structure was prepared by chemical corrosion. After the inner surface treatment, the heat transfer capability of the heat pipe was improved by 80%, and the thermal resistance was decreased by more than 44%. Zhong et al.^51^ reported an aluminum flat heat pipe, with multiple parallel microchannels for solar collector applications. HFE-7100 (C_4_F_9_OCH_3_) was then used as the working fluid. Moreover, the maximum heat transport capability was 40 W, with an optimal filling ratio of 25%. Although a lot of work has been devoted to improve the heat transfer performance of aluminum heat pipes, it is still a challenge to fabricate an aluminum powder-sintered wick structure in an aluminum heat pipe. The reason is the presence of a layer of aluminum oxide on the surfaces of the aluminum particles. It has, therefore, become necessary to develop a new approach to construct an aluminum wick structure with high thermal properties, i.e., to enhance the heat transfer capacity of the heat pipe.

In the present study, a micro-grooved aluminum flat heat pipe has been designed and fabricated by using a 3D-printing technology. The pipe, with its wick structure, was prepared by using a selective laser melting technology (which is a typical 3D printing technology). The wick structure of this 3D-printed flat heat pipe consists of sintered aluminum particles and designed micro-grooves. The role by the sintered aluminum particles is to increase the inner surface roughness, and the designed micro-grooves will further enhance the capillary force. Thus, the heat transfer performance of the heat pipe has become largely improved. Furthermore, acetone was selected as the working fluid, and the influence of the amount of working fluid on the heat transfer performance of the heat pipe was also investigated. As a result in the present study, a 10% load of the working fluid was found to give the lowest thermal resistance and the highest effective thermal conductivity. This specific load was also found to be able to maintain an excellent heat transfer performance in both vertical and horizontal operation modes. Our developed and constructed 3D-printed aluminum heat pipe was also tested in an LED system to provide an efficient thermal management. This test showed that the heat pipe will not only keep the LED working properly but will also prolong its service lifetime.

## Results and discussion

**Figure 1:**
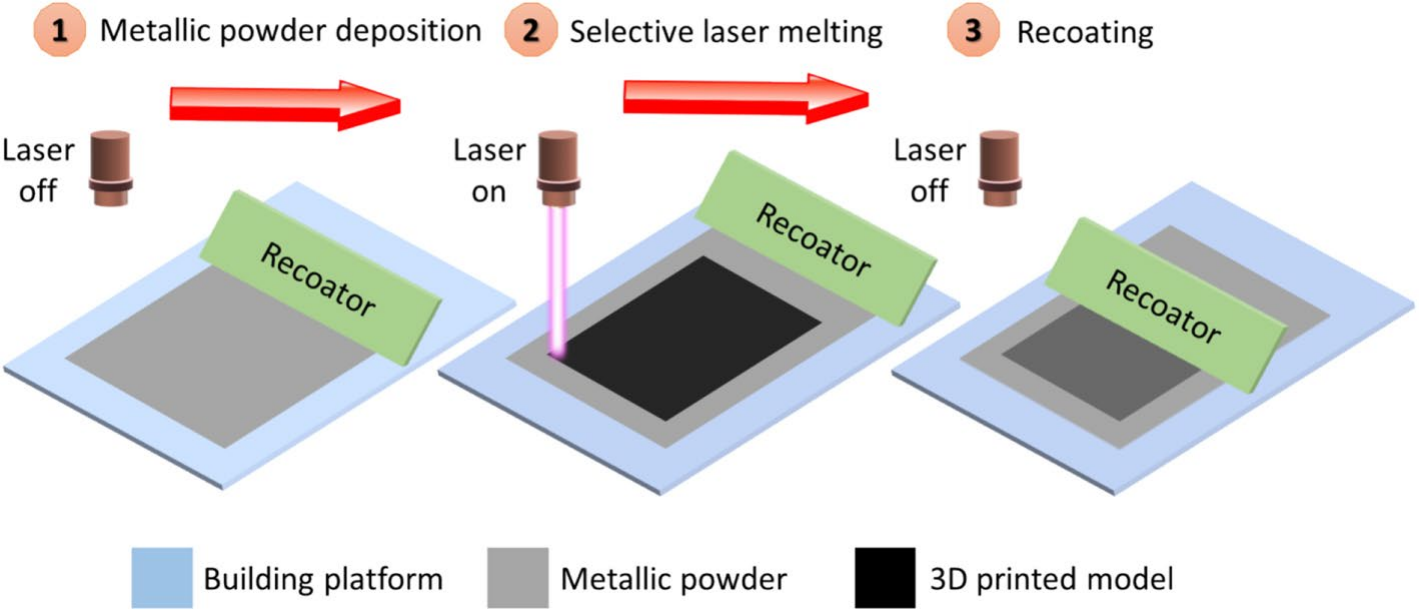
Fabrication process for 3D printing of a flat heat pipe.

A 3D-printed flat heat pipe has in the present work been fabricated by using a selective laser melting technology, which is a specific 3D printing technique. The fabrication process of the flat heat pipe is demonstrated in Fig. 1. A building platform was first covered with a thin layer of a metallic powder (step 1 in Fig. 1). A specific part of this layer was, thereafter, melted and fused by using a high-power laser (step 2). As a third step, the building platform was moved down a little distance, and another layer of metallic powder was formed by using a recoater. In addition, the laser did selectively melt and solidify the metallic powder. Steps 2 and 3 were, thereafter, repeated in a layer-by-layer process until the metallic layer had the shape of the desired 3D object (i.e., the flat heat pipe).

**Figure 2:**
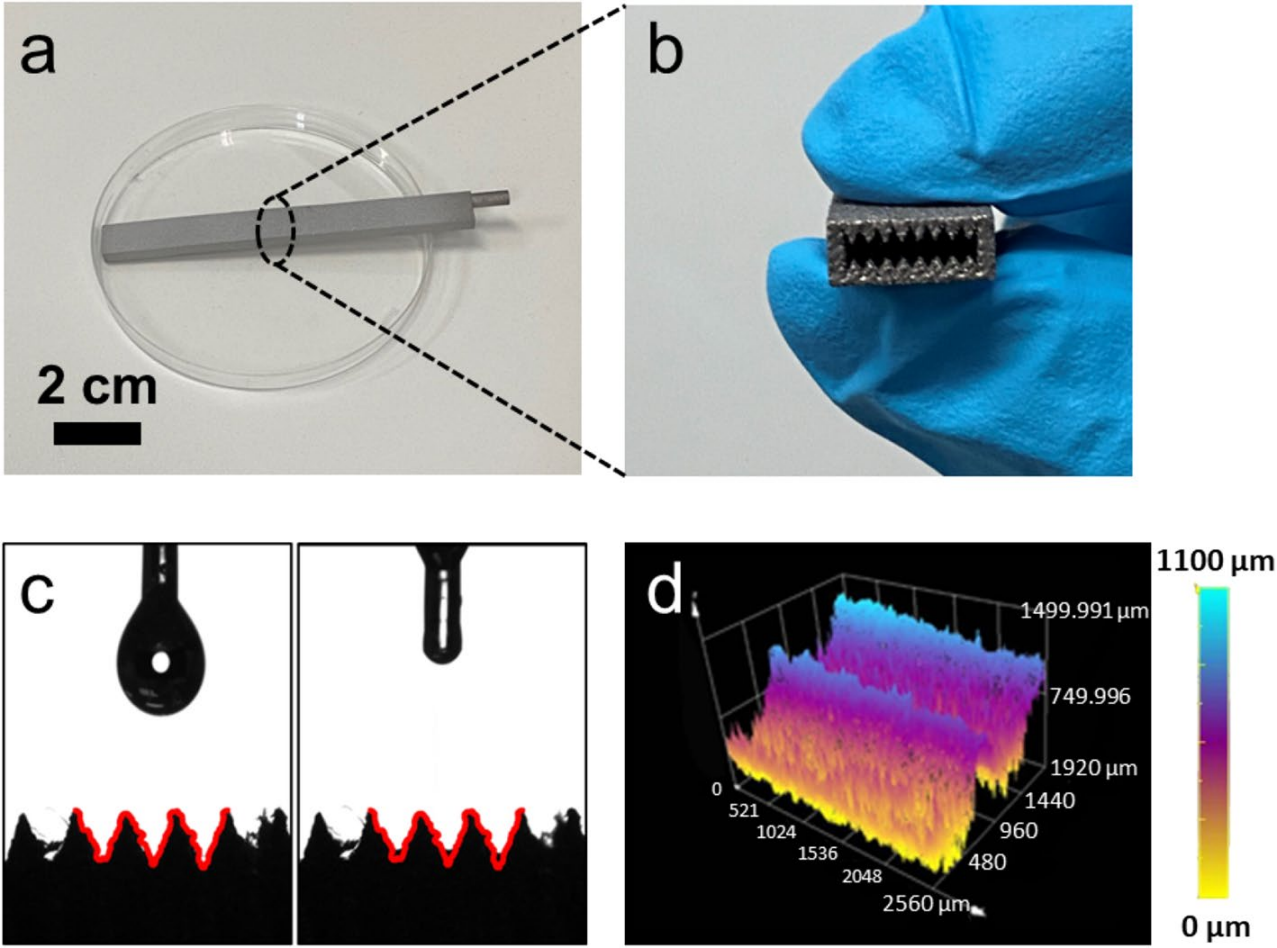
Characterization of the inner walls of a 3D-printed flat heat pipe with a wick structure: (**a**) image of the 3D-printed flat heat pipe; (**b**) cross section of the 3D-printed flat heat pipe, with its many micro-grooves; (**c**) contact angle measurements (before and after the attachment of a droplet of acetone onto wick structure); (**d**) 3D optical microscopic image showing the wick structure.

Figure 2a shows a picture of a flat heat pipe that has been prepared by using the above presented 3D printing technology. It is made of aluminum (Al) and of size 100 mm (length) × 10 mm (width) × 4 mm (height). To enhance the capillary force on the inner walls of the pipe, we have designed and fabricated sharp V-shaped grooves on the upper and lower walls (see Fig. 2b). The width and height of these grooves are 0.8 mm and 1 mm, respectively. These micro-grooves are expected to provide a sufficient capillary force to facilitate the pumping of the working fluid back to the evaporator section. They, therefore, play a significant role in the heat transfer performance of the heat pipe.

To characterize the wetting performances of the wick structure in the 3D-printed flat heat pipe, the pipe was cut into two halves (lengthwise). The wettability was, thereafter, determined by using an acetone droplet and a contact angle measurement instrument. As can be seen in Fig. 2c, the acetone contact angle is almost zero onto the micro-grooved surface. It was found that once an acetone droplet attached the inner surface of the 3D-printed flat heat pipe, it quickly spread out on the surface of the wick structure—in less than 0.1 s. The excellent wettability was mainly attributed to the sintered aluminum particles, which provided a rough inner surface of the heat pipe. The roughness of this wick structure was also measured by using a 3D optical microscopy (as shown in Fig. 2d). The resulting image reveals the existence of micro-grooves with heights slightly larger than 1 mm.

**Figure 3:**
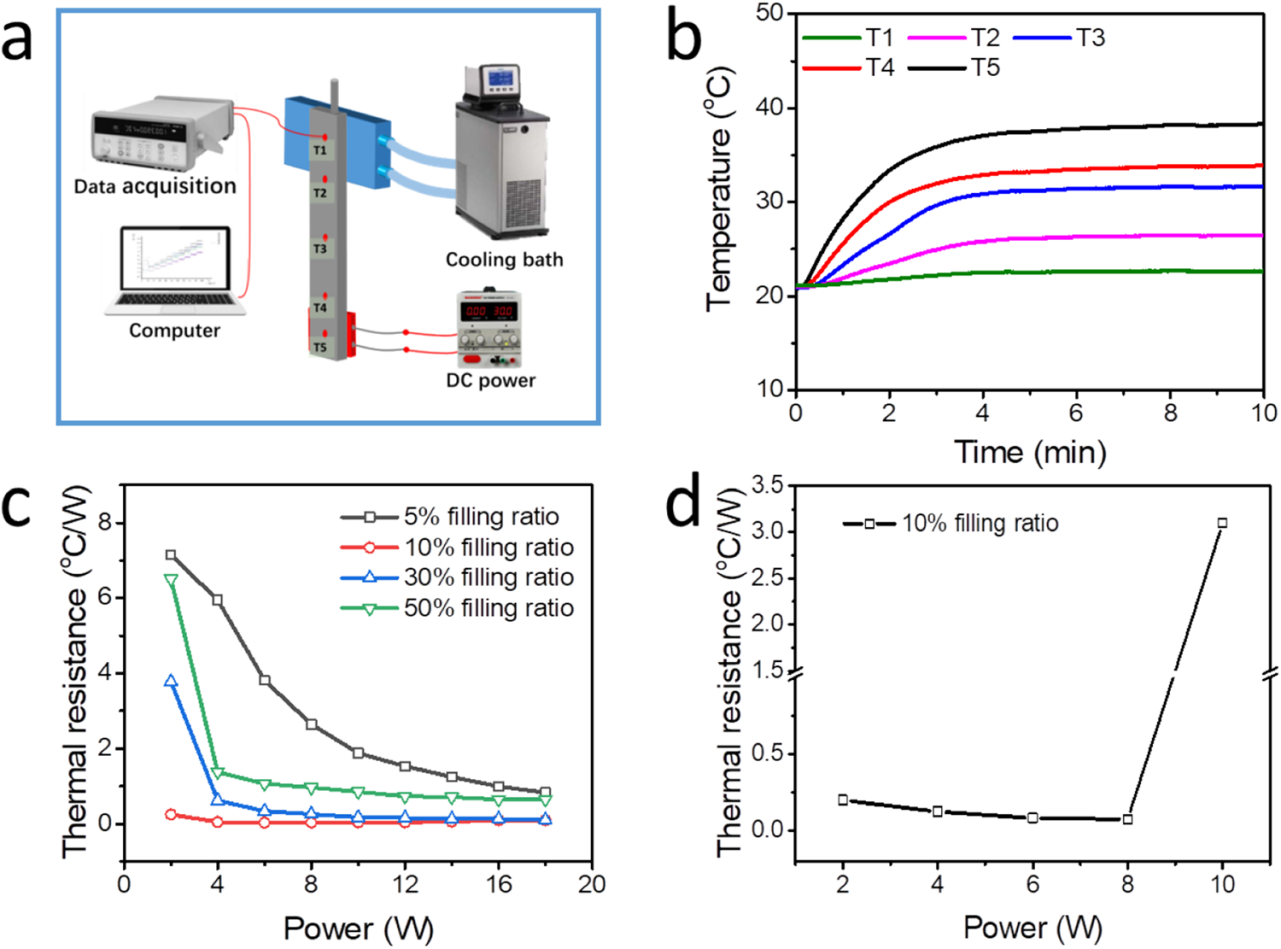
Experimental setup, and heat transfer performance, of the 3D-printed flat heat pipe: (**a**) schematics of the experimental setup; (**b**) temperature evolution of the 3D-printed flat heat pipe at a power of 2 W (at different positions along the heat pipe; T1–T5); (**c**) thermal resistance evolution of heat pipes loaded with different filling ratios (5%, 10%, 30% and 50%) of working fluid (in the vertical operation mode); (**d**) thermal resistance evolution of a heat pipe in the horizontal operation mode (for a 10% filling ratio).

After the construction and characterization of the flat heat pipe, a back-filling method^16–19^ was used to fill the pipe with liquid acetone. The heat pipe was, then, first pumped to a vacuum below 5 Pa, and then backfilled with different volumes of acetone. The filling ratio was defined as the ratio between the volume of loaded working fluid (i.e., acetone in this case) and the inner space of the flat heat pipe. Four different filling ratios were used (5%, 10%, 30%, and 50%) and the main purpose was to investigate the influence of filling ratio on the heat transfer performance. The experimental setup for evaluating this heat transfer can be seen in Fig. 3a. A ceramic heater, with a size of 10 mm × 10 mm, was positioned onto the evaporator section of the 3D-printed heat pipe, and connected to an adjustable DC power (i.e., for input power). Moreover, a cooling block, with a temperature of 20 °C, was positioned onto the condenser section (i.e., to receive the conducted thermal energy). To minimize any heat loss, the whole heat pipe was wrapped by a porous thermal insulation material. Furthermore, to evaluate the heat transfer performance of the heat pipe, five thermocouples were equally distributed along the pipe (from the evaporator section to the condenser section) (see Fig. 3a).

During the experiment, the input power was increased by adjusting the DC power from 2 to 18 W (in steps of 2 W). The measurements lasted for 10 min at each DC power, to ensure that the heat pipe had reached a steady working state. The thermal resistance of the heat pipe, *R,* was calculated by using the following equation:

1 $$R = \frac{{T_{e} - T_{c} }}{q}$$

where $q$ is the input power, $T_{e}$ is the temperature of the evaporator section, and $T_{c}$ is the temperature of the condenser section.

The temperature evolution profile of the 3D-printed flat heat pipe in the vertical operation mode (with 5% filling ratio and an input power of 2 W) is shown in Fig. 3b. The measured temperatures, at all five measuring points, did at first increase gradually and then remain constant over time. This is an indication of the fact that the heat pipe has reached a steady working state. Hence, the thermal resistance, *R*, can be calculated by using Eq. (1).

The level of working fluid has a strong impact on the heat transfer performance of the heat pipe. Generally, when the heat pipe is filled with too much working fluid, the condenser section will be over-flood, resulting in a poor thermal performance. When the amount of working fluid is too low, there is not enough fluid in the evaporator section to absorb the input power to complete the whole thermodynamic cycle. Thus, there is an optimized filling ratio for the heat pipe. Figure 3c shows the thermal resistance of the flat heat pipe when loaded with different percentage of working fluid (5%, 10%, 30% and 50%) in a vertical operation mode. As shown in this figure, the thermal resistance drops at a higher input power. Due to the lack of sufficient acetone to cover the cycling loop, the heat pipe loaded with 5% filling ratio has a larger thermal resistance. Increasing the filling ratio to 30% will reduce the thermal resistance, but when filling ratio is increasing to 50%, the thermal resistance of heat pipes again increases. That is because that when the heat pipe loads too much working fluid, the excessive working fluid will block the vapor flow and cover the condensation area, thereby limiting the normal thermodynamic cycle^25^. The best heating performance, with the lowest thermal resistance, is observed for the situation with a filling ratio of 10%. In fact, the thermal resistance is less than 0.1 °C/W when the input power is 18 W. Therefore, a 10% filling ratio has been considered as the optimum filling ratio for this specific 3D-printed flat heat pipe. Following the law of Fourier, the effective thermal conductivity of the heat pipe, $k$, can be calculated by using the following equation:

2 $$q = kA\frac{\Delta T}{d}$$

where $A$ is the cross section area of the flat heat pipe, and $d$ is the distance between the evaporator section and condenser section. According to Eq. (2), the average effective thermal conductivity of the 3D-printed flat heat pipe is 20,803 W/m K for an increase in input power from 2 to 18 W.

To further evaluate the heat pipe in practice, the heat transfer performance was also studied for the pipe positioned horizontally. The purpose with this experimental setup was to eliminate the influence of gravity. Figure 3d shows the variation in thermal resistance for a 10% filling ratio and the heat pipe in a horizontal operation mode. As can be seen in Fig. 3d, the thermal resistance does gradually decrease as the input power increases from 2 to 8 W. However, the thermal resistance increases sharply when increasing the input power to 10 W. This means that the operating limit has been reached. It is because that in the absence of gravity effects, there is no enough force to pump the working fluid back to the evaporator section, resulting in the operating limit. In summary, for an input power between 2 and 8 W, the 3D-printed flat heat pipe presents good thermal performance with low thermal resistance. No matter if the vertical, or horizontal, operation mode is in use.

**Figure 4:**
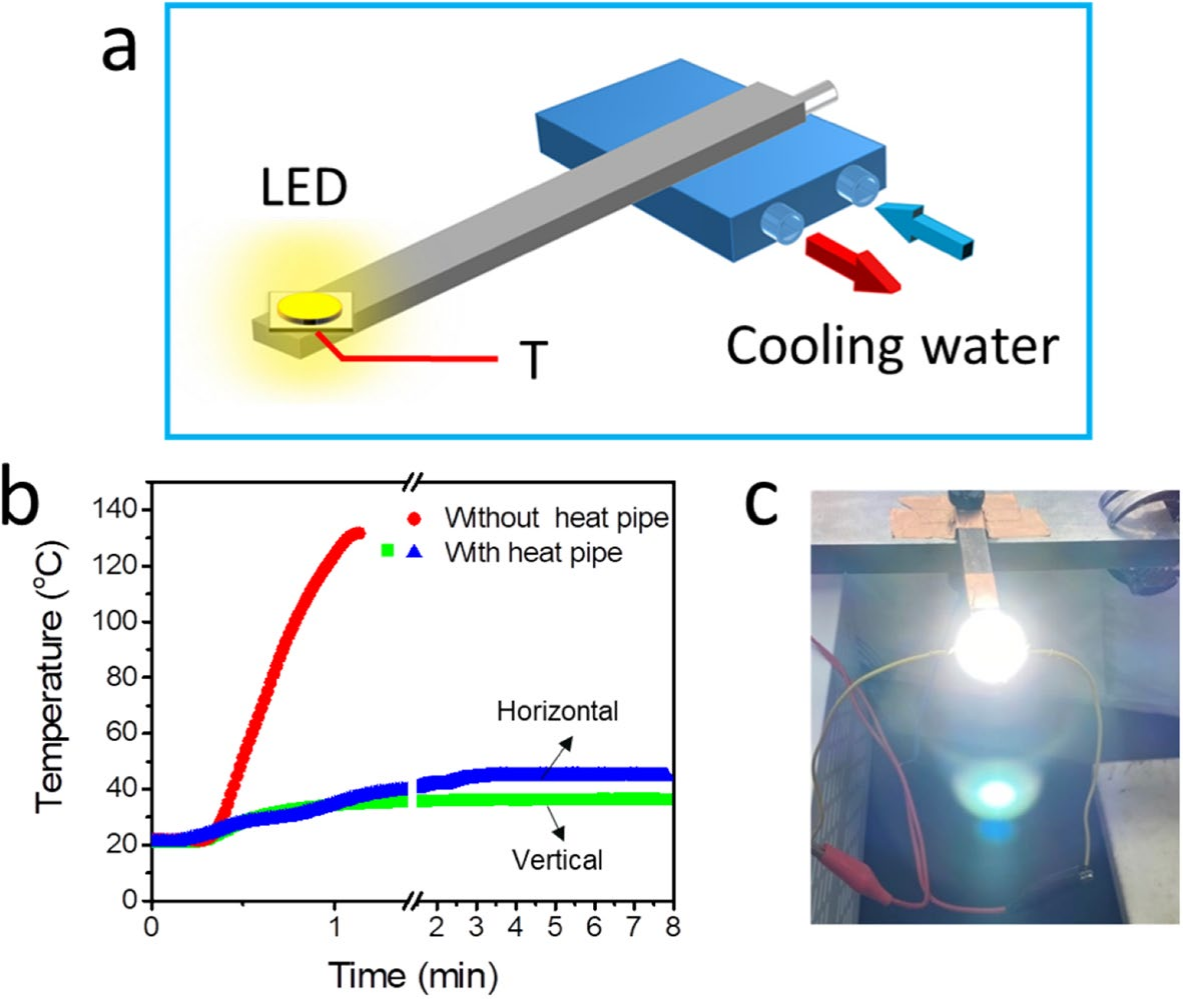
Thermal management of an LED by using the 3D-printed flat heat pipe: (**a**) schematics of the experimental setup; (**b**) temperature evolution of the LED (over time); (**c**) a photograph showing that the LED can light up with the assistance of the heat pipe.

Due to the high heat transfer performance of the 3D-printed flat heat pipe, its use for thermal management of high-power LEDs has also been studied. As can be seen in Fig. 4a, a LED chip (with a heat generation of 6 W) was then connected to one end of the heat pipe. The other end of the heat pipe was connected to a cooling block, with a constant temperature of 20 °C (see Fig. 4a). The heat generated by the LED was then transferred through the 3D-printed flat heat pipe and became dissipated by the cooling block. In addition, a thermocouple was placed on the back of the LED to monitor the real-time temperature change of the LED.

The resulting temperature evolution profile of the LED is shown in Fig. 4b. When the LED was lit up without the presence of the 3D-printed flat heat pipe, the LED temperature quickly reached a temperature of 130 °C (within 1 min), which destroyed the LED. However, when the LED was connected to the heat pipe, the temperature quickly reached the steady working state at 35 °C, which prolonged the service lifetime of the LED. In addition, the effect of gravity on the thermal performance of the heat pipe was also investigated. When the heat pipe was horizontally positioned, the temperature of the LED was maintained at 40 °C, which is slightly higher than that in the vertical operation mode (see Fig. 4b). In the horizontal operation mode, only the capillary force induced by the wick structure drives the working fluid back to the evaporator section. In the vertical operation mode, both the gravity force and capillary force pump the working fluid back to the evaporator section, thereby resulting in a better thermal performance. The functioning of the LED, when connected to the 3D-printed flat heat pipe, is shown in Fig. 4c. It has here been shown that the LED is able to work normally with the operation of the 3D-printed heat pipe.

## Conclusions

An aluminum flat heat pipe, with micro-structured inner walls, has here been constructed by using a 3D printing technology. This technology not only enables the complex wick structure within the heat pipe, but also realizes a simplified production of heat pipes. The designed inner walls of the heat pipe, with their micro-grooves and sintered rough inner surface, will offer a sufficiently large capillary force to pump the working fluid back to the evaporator section. Acetone was selected as the working fluid, and the effect of various fluid filling ratios, on the heat transfer performance (of the heat pipe), was investigated. The results show that a 10% working fluid gives an excellent heat transfer performance with low thermal resistance. It was also shown that the 3D-printed flat heat pipe can work in both vertical and horizontal operation modes. Furthermore, it was demonstrated that the 3D-printed flat heat pipe can be applied for the thermal management of LEDs. Other types of applications include the CUP, high power electronics, battery thermal management and many other systems that require heat dissipation.

## Methods

### Materials

AlSi10Mg powder was ordered from CNPC POWDER Co., Ltd. Ar (99.99%) was purchased from Dalian Date Gas Co., Ltd. Acetone was ordered from Aladdin Reagent (Shanghai, China). The ceramic heater (10 mm × 10 mm) was bought from Beijing Youpusi Technology Co., Ltd. Thermal grease was purchased from Shanghai Zihong Electronics Co., Ltd.

### Fabrication of 3D-printed flat heat pipes

AlSi10Mg powder with a size of 20 µm was chosen as a raw material. The container and inner wick structure of the 3D-printed flat heat pipe was fabricated by a 3D metal printer (EOS M290). Ar was used as protective gas during the synthetic process. The laser power, scan speed and hatch distance were 400 W, 300 mm/s and 0.10 mm, respectively. The layer thickness was kept constant at 0.03 mm. Four flat heat pipes were printed in a 3D-printed process. The four heat pipes were arranged in a line, and placed in the middle of the building chamber. The as-prepared 3D-printed flat heat pipe was placed at an oven with 200 °C for 2 h in order to eliminate internal stress. We adopted a back-filling method to fill the working fluid into the 3D-printed flat heat pipe. Acetone was used as the working fluid. The heat pipe was connected to our previous charging system by a silicon tube. We first evacuated the 3D-printed flat heat pipe to a vacuum of 5 Pa, backfilled with different volumes of acetone working fluid, and then fully sealed.

### Measurement and characterization

The 3D optical microscope images of inner wick were measured by a 3D digital microscope (Keyence VHX-S50). The contact angle of the wick structure was characterized by a contact angle measuring instrument (FM40Mk2 EasyDrop, KRUSS GmbH). Temperature distribution of the 3D printed flat heat pipe was collected by K-type thermocouples (Omega SMPW-TT-K, resolution ∼ 0.1 °C) which were connected to a multichannel data acquisition system (Agilent 34972A, Agilent Technologies Inc.). To evaluate heat transfer performance of the heat pipe, a cold block was attached to the heat pipe at the condenser section, and a cooling bath (Julabo Bilon Equipment) provided circulated running water with a consistent temperature of 20 °C. The ceramic heater and cold block were bonded with thermal grease on the back surface of the 3D printed flat heat pipe.
